# Regulatory Cross-Talks and Cascades in Rice Hormone Biosynthesis Pathways Contribute to Stress Signaling

**DOI:** 10.3389/fpls.2016.01303

**Published:** 2016-08-26

**Authors:** Arindam Deb, Rumdeep K. Grewal, Sudip Kundu

**Affiliations:** ^1^Department of Biophysics, Molecular Biology and Bioinformatics, University of CalcuttaKolkata, India; ^2^Computational Systems Biology Group, Center of Excellence in Systems Biology and Biomedical Engineering, University of CalcuttaKolkata, India

**Keywords:** hormone biosynthesis, transcriptional regulation, cis-regulatory element, cross-talk, Magnaporthe, drought

## Abstract

Crosstalk among different hormone signaling pathways play an important role in modulating plant response to both biotic and abiotic stress. Hormone activity is controlled by its bio-availability, which is again influenced by its biosynthesis. Thus, independent hormone biosynthesis pathways must be regulated and co-ordinated to mount an integrated response. One of the possibilities is to use cis-regulatory elements to orchestrate expression of hormone biosynthesis genes. Analysis of CREs, associated with differentially expressed hormone biosynthesis related genes in rice leaf under *Magnaporthe oryzae* attack and drought stress enabled us to obtain insights about cross-talk among hormone biosynthesis pathways at the transcriptional level. We identified some master transcription regulators that co-ordinate different hormone biosynthesis pathways under stress. We found that Abscisic acid and Brassinosteroid regulate Cytokinin conjugation; conversely Brassinosteroid biosynthesis is affected by both Abscisic acid and Cytokinin. Jasmonic acid and Ethylene biosynthesis may be modulated by Abscisic acid through DREB transcription factors. Jasmonic acid or Salicylic acid biosynthesis pathways are co-regulated but they are unlikely to influence each others production directly. Thus, multiple hormones may modulate hormone biosynthesis pathways through a complex regulatory network, where biosynthesis of one hormone is affected by several other contributing hormones.

## 1. Introduction

**Figure 1:**
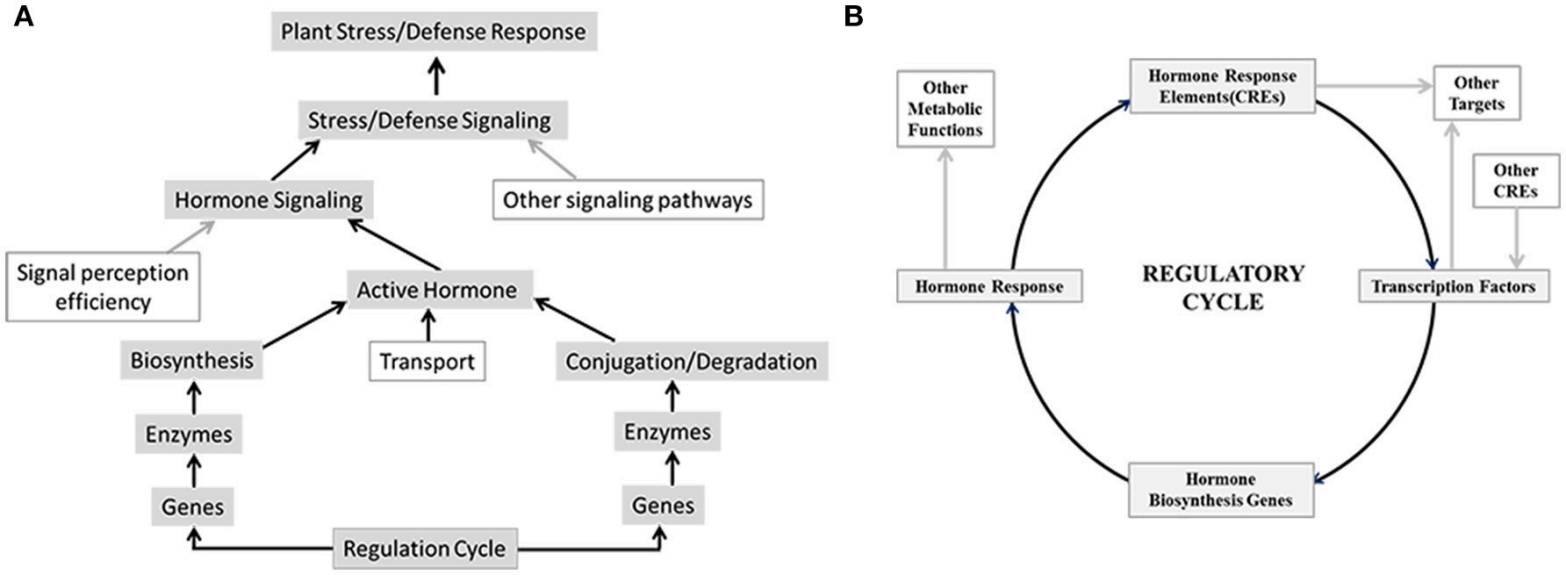
**Overview of factors that may regulate hormone signaling and biosynthesis, which leads to plant stress and defense response**. **(A)** Hormone signaling brought about by biological active hormone depends on their availability which is controlled by level of their synthesis, transport and conjugation or degradation as well as efficiency of signal perception and transduction. Modulations in any of these have direct impact on downstream stress and defense responses. **(B)** Transcription factors bind to cis-regulatory elements (CRE) in the promoters of hormone biosynthesis genes and regulate hormone biosynthesis and consequently hormone signaling and response. Conversely promoters of transcription factor genes may harbor hormone responsive CREs that control transcription of these genes in response to hormone signaling.

Plants being sessile creatures are forced to adapt to various adverse conditions to successfully complete their life-cycles. A repertoire of complex signaling systems was acquired by plants to respond to physiological and environmental cues. Phytohormones are one of the key mediators that afford plants the ability to rapidly respond to external cues by adjusting their metabolism. Previous research efforts have revealed importance of individual hormones in plant development and stress response (Creelman and Mullet, 1997; Yamaguchi, 2008; Zhao, 2010). Biological activity of any hormone depends on its availability which is controlled by level of its synthesis, transport and conjugation or degradation as well as efficiency of signal perception and transduction. Modulations in any of these have direct impact on downstream hormone responses (Figure 1A). Moreover, activities of various hormones have been shown to be overlapping; and cross-talk among different hormonal response pathways is a well-established phenomenon (Gazzarrini and McCourt, 2003; Robert-Seilaniantz et al., 2011; Vanstraelen and Benková, 2012). Hormonal interactions provide an additional level of complexity in regulation of plant response to internal and external cues as well as render a feedback mechanism to balance system robustness and dynamicity. Thus, the final response of a plant is not determined by activity of a single hormone but rather by a complex network where activity of a hormone is also modulated by other contributing hormones.

**Figure 2:**
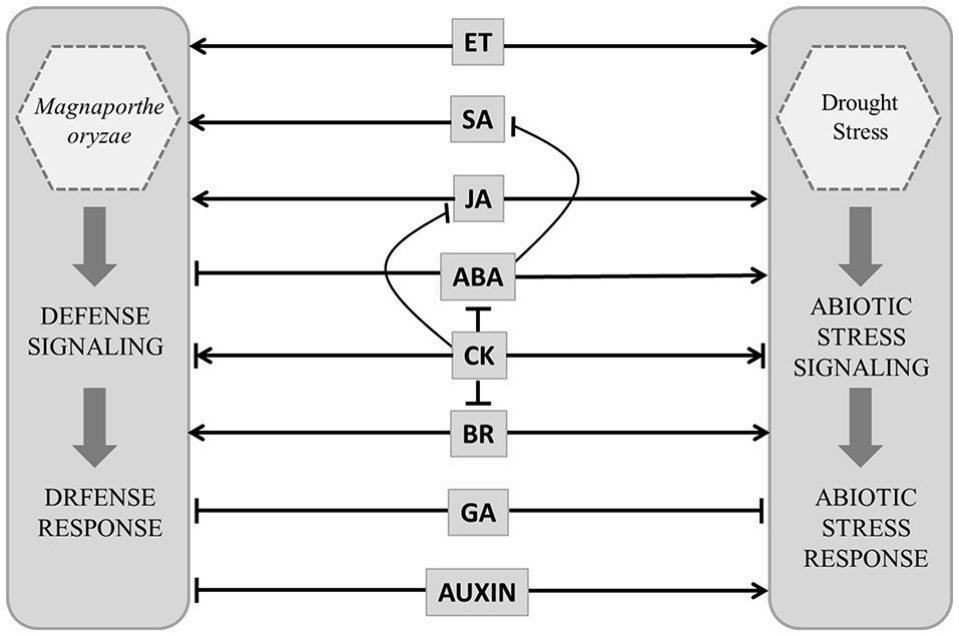
**Model showing probable hormone defense (***Magnaporthe oryzae*** infection) and stress (drought) networking in ***Oryza sativa*****. Salicylic acid (SA), jasmonic acid (JA) ethylene (ET), brassinosteroid (BR) and abscisic acid (ABA) interact positively or negatively depending upon stress conditions. ABA is antagonistic to SA and suppresses disease resistance but promotes abiotic stress tolerance. Gibberellic acid (GA) suppress stress responses. Auxin suppress defense against pathogen but augments abiotic stress tolerance. Cytokinin (CK) interacts negatively with ABA, JA and BR. Positive and negative regulatory actions are indicated by arrows and lines with bars, respectively.

In rice both disease resistance and abiotic stress responses appear to be controlled by a complicated hormone signaling network. Salicylic acid (SA) and jasmonic acid (JA) promote resistance against pathogens (Figure 2). Ethylene (ET) may have negative or positive impact on disease resistance depending upon pathogen lifestyle (De Vleesschauwer et al., 2013). Abscisic acid (ABA), JA and ET interact positively or negatively depending upon stress conditions to promote stress responses. ABA is known to be antagonistic to SA and suppresses disease resistance but promotes abiotic stress tolerance (Kohli et al., 2013). The interactions of growth hormones are equally complex e.g., gibberellic acid (GA) is known to suppress stress responses through its antagonistic interaction with both ABA and JA (De Vleesschauwer et al., 2013). Auxin is also known to suppress defense against pathogen but augments abiotic stress tolerance (Kohli et al., 2013). Brassinosteroid (BR) through its interaction with stress hormones as well as growth hormones plays important role in biotic and abiotic stress response. Role of cytokinin (CK) in stress management is complicated and condition specific. CK interacts negatively with ABA, JA, and BR and promotes stress tolerance by delaying stress response (Figure 2). It is depleted from tissue exposed to prolonged stress, but accumulates during stress of short duration. Despite this cross-talk the metabolic pathways controlling individual hormone production are specific and apparently non-redundant. This poses the questions about regulation of independent hormone biosynthesis pathways and the possible ways in which multiple hormone production pathways may be modulated resulting in an integrated response.

Regulation of hormone biosynthetic pathways at both transcriptional and post transcriptional level has been well documented (Xiong and Zhu, 2003; Frigerio et al., 2006; Argueso et al., 2007), however modulations of these pathways in an integrated way remains unclear. One of the possibilities may be to use common transcription regulators for several hormones thereby controlling the transcription of genes related to more than one hormone biosynthesis at the same time. Transcriptional control of genes is brought about by cis-regulatory elements (CRE), located in promoters of target genes, and their corresponding transcription factors. As the promoters may harbor multiple CREs, gene expression depends upon binding of corresponding transcription factors and the specific combinatorial logic among them (Figure 1B). CRE mediated regulation has the potential to produce gene expression patterns which may be tissue and/or condition specific. Several computational approaches intended to understand the CRE mediated combinatorial regulation at the promoter regions of genes (Pilpel et al., 2001; Sudarsanam et al., 2002; Kato et al., 2004; Yu et al., 2006; Murakami et al., 2008; Deb and Kundu, 2015) are available. In present work, we applied the existing computational approaches to study possible CRE mediated regulation of hormone biosynthetic pathways in rice leaf and the dynamics of these regulation under *Magnaporthe oryzae* attack (causal organism of blast disease) and drought stress. Results of this work allowed us to map the CRE-mediated regulation of differentially up-regulated rice hormone biosynthesis (HB) genes under blast and drought conditions. Through analysis of CREs associated with differentially expressed HB genes we were able to develop insights about effect of different hormones and stress conditions on regulation of all HB pathways. We were also able to identify key transcription factors for CRE elements that integrate and modulate these pathways.

## 2. Materials and methods

### 2.1. Promoter sequence data

Promoter sequence data (1 kb upstream) of rice loci is collected from Rice Genome Annotation Project, version 6.1 (Ouyang et al., 2007). RepeatMasker, version open-4.0.2 (Smit etal., 2013–2015) is used to mask interspersed and simple repeats in the promoter sequences. This filtering restricts unwanted discovery of CREs into the stretches of repeated DNAs present in the promoters.

### 2.2. Cis-regulatory element data

The information of CREs is collected from PLACE (Plant cis-acting regulatory element) database (Higo et al., 1999). This initial dataset is screened to fetch only the unique entries. Signal Scan (Prestridge, 1991) tool is used to fetch the occurrences and positions of the CREs in the promoters.

### 2.3. Mapping plant hormone biosynthesis (HB) pathway genes

The information of rice metabolic pathways and their associated loci is collected from the RiceCyc database (v.3.3) (Jaiswal et al., 2006). The initial data is further screened to get the hormone biosynthesis pathways. All together information for 8 different plant hormone biosynthesis pathways (Auxin, ABA, BR ,CK, ET, GA, JA, and SA) was obtained.

### 2.4. Gene expression data

Gene expression data were collected from Gene Expression Omnibus (GEO) (Edgar et al., 2002) for Rice under two different conditions, blast infection (GSE7256) and drought (GSE26280). RMA (Robust Multiarray Average) normalization was performed using R and Bioconductor (Gentleman et al., 2004) and differentially up-regulated genes (log fold change >2; *p* < 0.05) from each experiment (4 dpi of GSE7256 and panicle stage leaf of GSE26280) were considered for further analysis.

### 2.5. Estimation of over-represented CREs in individual rice promoters

The over-represented CREs in the promoters can be obtained by different statistical tests. We used cumulative hypergeometric statistics (Hughes et al., 2000; Sudarsanam et al., 2002) (Equation 1) to determine the over-represented CREs in individual promoters of the respective genes.

(1) $$P_{CRE}=1-F(x,N,n,m)=1-\sum_{\;t=\;0}^{x}\frac{\left(\begin{array}{c} m\\ t \end{array}\right)\left(\begin{array}{c} N-m\\ n-t \end{array}\right)}{\left(\begin{array}{c} N\\ n \end{array}\right)}$$

Where, *N* is the total number of occurrences of all CREs in rice genome, *m* is the total number of occurrences of a considered CRE in the genome, *x* is the number of occurrences of that CRE in an individual promoter of a gene, *n* is the total number of occurrences of all CREs in that promoter and *t* is the summation index. Thus, *P*_*CRE*_ is the probability of occurrence of a considered CRE in a particular promoter. A particular CRE was considered as over-represented in a specific promoter if the respective *P*_*CRE*_ is < 0.05. This statistics was executed for all CREs in each HB locus promoter.

### 2.6. Estimation of CRE co-occurrence

In our previous work (Deb and Kundu, 2015) a unique methodology has been introduced to identify the significantly co-occurring CRE pairs in the rice promoters and to develop different level of networks using them; the same methodology is applied in the present study. We computed the co-occurrence tendency in terms of *COR* value for all possible CRE pairs present in the promoters of differentially up-regulated HB genes for each condition (blast and drought). A series of filtering steps (Deb and Kundu, 2015) were used to extract out only the significantly co-occurring CRE pairs.

### 2.7. Construction of networks: locus-locus co-enriched CRE network and CRE co-occurrence network

We constructed locus-locus co-enriched CRE networks and CRE co-occurrence networks for each condition (blast and drought) separately. The locus-locus co-enriched CRE network was developed using the up-regulated HB genes and the over-represented CREs at their promoters. Each node of the network was specified by individual locus and two loci were interconnected if they have at least one common over-represented CRE.

Individual CRE co-occurrence network was constructed using the significantly co-occurring CRE pairs where nodes represent individual CRE and two CREs were connected if they significantly co-occur with each other (Deb and Kundu, 2015). We intended to find the co-occurring CRE combinations in the form of cliques. Clique(*k*) is interpreted as a complete (fully connected) subgraph having *k* number of nodes. In CRE co-occurrence network, a clique represents a combination of co-occurring CREs having statistically significant co-occurrence relationship with every other CREs of the same clique. CFinder (Adamcsek et al., 2006) was used to identify the cliques in each network.

## 3. Results

### 3.1. CREs in up-regulated hormone biosynthesis genes under blast and drought

We found 37 HB loci in blast infected leaf and 26 HB loci in leaf under drought stress to be up-regulated. In case of blast dataset (GSE7256), genes associated with Gibberellin biosynthesis were absent. Similarly Salicylic acid biosynthesis associated genes were absent from drought dataset (GSE26280) (Table S1). This indicates that SA pathways are unlikely to have any role in case of drought stress whereas JA pathway has important implications.

We estimated over-represented CREs in individual promoters of these two sets of loci. On an average 8 CREs (8.2 in blast and 7.8 in drought) were over-represented in individual promoters. Promoter of ABA biosynthesis locus *Os*07*g*05940.1 was associated with highest number of CREs, sixteen in number. This locus was common to both blast and drought datasets. Lowest number of CREs was associated with JA biosynthesis locus *Os*06*g*11210.1, it had a single CRE element (Table S1).

### 3.2. CRE co-occurrence networks of hormone biosynthesis genes under blast and drought

The co-occurrence tendency for all possible CRE pairs present in the promoters of differentially up-regulated HB genes for each condition (37 for blast and 26 for drought) were calculated and significantly co-occurring pairs based on the filtering steps (Deb and Kundu, 2015) were extracted and used to generate two separate CRE co-occurrence networks. Network for blast consisted of 137 nodes (CREs) and 296 edges (co-occurring relationship) whereas, that for drought consisted of 122 nodes and 232 edges. In case of blast condition, the co-occurrence network shows clustering coefficient 0.16, network density 0.03 and characteristics path length 3.11. On the other hand, the drought related network has clustering coefficient 0.06, network density 0.03 and characteristics path length 3.59.

**Figure 3:**
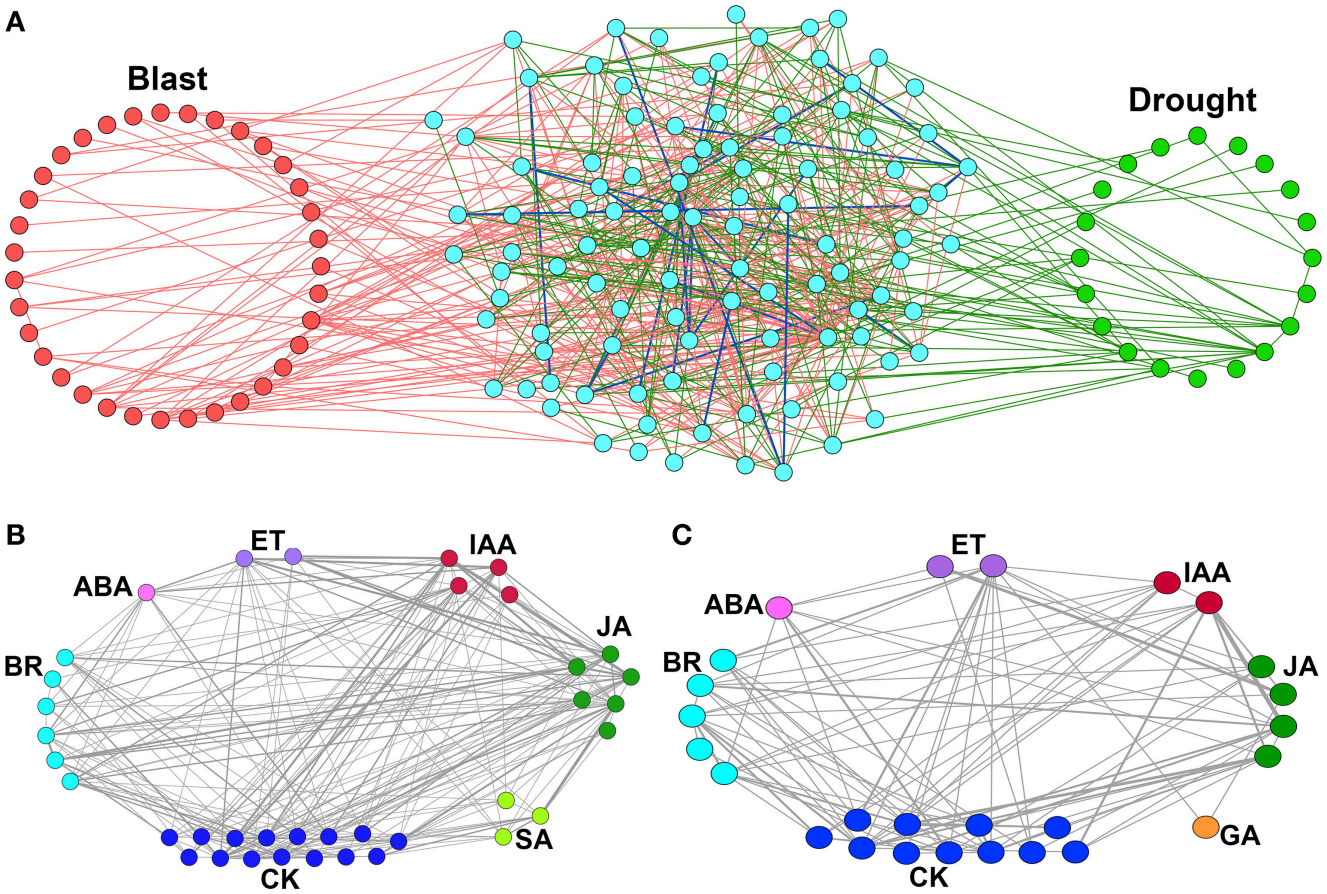
**Merged CRE co-occurrence network and locus-locus co-enriched CRE networks of hormone biosynthesis genes up-regulated under ***Magnaporthe oryzae*** infection and Drought**. **(A)** Merged CRE co-occurrence network of hormone biosynthesis genes up-regulated under *Magnaporthe oryzae* infection—Blast and Drought. Nodes (individual CREs) explicitly found in the Blast are highlighted (red) in the left, while those found in the Drought are highlighted (green) in the right hand side of the network. Nodes in the middle (cyan) are found in both Blast and Drought. Pink and bottle green color of edges imply that a significant co-occurrence is found between the two connected CREs explicitly in Blast and Drought respectively. A blue edge implies that the two connected CREs significantly co-occur in both the conditions. **(B,C)** Locus-locus co-enriched CRE networks for up-regulated hormone biosynthesis (HB) genes. **(B)** *Magnaportheoryzae* infection and **(C)** during drought stress. Each node of the network represents individual locus and two loci are interconnected if they have at least one common over-represented CREs. The edge weight represents the number of common over-represented CREs among them.

When we merged these two networks, a relatively large number of nodes were found to be common (102), whereas, number of common interacting edges were relatively small in number (24) (Figure 3A). This indicates that though common CREs are present in the promoters of both blast and drought up-regulated HB genes, their co-occurring interaction patterns differ from condition to condition. This further indicates a possibility of presence of unique combinations of transcription factors that regulate the biosynthetic genes at two different stresses.

### 3.3. Locus-Locus co-enriched CRE network

The locus-locus co-enriched CRE network was developed using the up-regulated HB genes having over-represented CREs at their promoters. Each node of the network represents individual locus and two loci are interconnected if they have at least one common over-represented CREs. Thus, the interacting edges represent the regulatory similarities among the promoters (loci) where the edge weight shows the number of common over-represented CREs among them. Interestingly we found that loci of same HB pathways as well as of different HB pathways share abundant edges. This indicates that different HB pathways may have common regulators (Figures 3B,C).

### 3.4. Cliques of CRE co-occurrence networks

Clique of CREs is a combination of co-occurring CREs i.e., each CRE has significant co-occurrence relationship with other members of the same clique. Clique (*k* = 3–8) analysis was performed on CRE co-occurrence network for each condition. In case of blast and drought 76 and 29 cliques were observed, respectively. Twenty one cliques in case of blast and a single clique in case of drought were found to encompass all the HB pathways. Whereas 55 cliques in blast and 28 cliques in drought were found to be present only in loci of particular HB pathways. It was interesting to note that in case of Blast we did not find any clique which are exclusive to a single HB pathway; in case of drought we found two (d0:[CACTFTPPCA1, GTGANTG10, PROXBBNNAPA] and d14:[CPBCSPOR, CACTFTPPCA1, PROXBBNNAPA]) exclusive to cytokinin biosynthesis genes. Under blast infection 7 cliques were associated with all 37 up-regulated HB loci, whereas under drought condition a single clique was associated with 25 (out of 26) up-regulated HB loci and another was associated with 22 up-regulated HB loci. These 9 cliques were composed of 10 CREs of which 6 were common to both blast and drought loci (Table 1). The common CREs are binding sites for GATA, GT1, RAV1, ARR1, DOF, and WRKY71 transcription factors of these GT1, RAV1, and WRKY71 are reported to be associated with stress response. GT1 influences SA inducible expression of pathogenesis related—PR proteins (Buchel et al., 1999). RAV1 is a positive regulator of leaf senescence (Woo et al., 2010) and WRKY71 is related to both biotic and abiotic stress response (Xie et al., 2006; Liu et al., 2007). These observations indicate that transcription factors that bind to these CREs may act as master regulators of defense and stress response (Table 1).

**Table 1:** **List of CREs and corresponding transcription factors (TF) associated with up-regulated hormone biosynthesis loci under blast (***Magnaporthe oryzae*** infection) and drought stress**.

**CRE name**	**Corresponding TF**	**Number of up-regulated loci**	
		**Blast**	**Drought**
ARR1AT	ARR1	37	0
DOFCOREZM	DOF	37	0
WRKY71OS	WRKY71	37	22
GATABOX	GATA	37	25
POLLEN1LELAT52	–	37	4
GTGANTG10	–	37	3
CACTFTPPCA1	–	37	14
RAV1AAT	RAV1	24	22
GT1CONSENSUS	GT1	0	25
GT1GMSCAM4	GT1	0	22

In blast infected leaves BR HB loci were most abundant in cliques (94.7%) followed by CK (93.7%), JA (85.5%) and SA (75.0%) (Figure S1). In case of drought SA up-regulated loci were not observed in expression data so were completely absent but BR (82.8%), CK (79.3%) and JA (86.2%) showed similar trend (Figure S1). Loci of ethylene biosynthesis pathway were found to be associated with 50 cliques of which 48 were associated with JA and 46 were associated with both JA and SA. This indicates the existence of some master transcription factors which regulate both ET and JA biosynthesis genes and all three (ET, JA, SA, HB genes).

## 4. Discussion

Expression analysis of HB pathway associated genes under *Magnaporthe oryzae* infection and drought stress, demonstrates that some genes are differentially up-regulated under both conditions whereas some are exclusively up-regulated in either condition. These findings indicated that differential regulation of hormone biosynthesis may contribute to stress response. Analysis of CREs in promoters of these up-regulated HB genes allowed us to extract insights about regulation of HB pathways and CREs (and/or transcription factors) that may integrate and modulate hormone biosynthesis.

### 4.1. Cytokinin conjugation is regulated by ABA

Role of cytokinins in regulating plant growth and development by promoting cell division and stimulating sink strength is well established. Recently evidence has accumulated demonstrating importance of CK in environmental stress response as well. Endogenous CK levels were reported to alter stress tolerance of plants and conversely stress conditions were found to change CK content in plants (Kudoyarova et al., 2007; Rivero et al., 2007; Albacete et al., 2008; Ghanem et al., 2008; Havlová et al., 2008; Werner et al., 2010; Nishiyama et al., 2011). Studies of stress response in CK-deficient plants showed that CKs negatively regulate stress signaling (Werner et al., 2010; Nishiyama et al., 2011). Down-regulation of CK active content is associated with prolonged drought and salt stress (Kudoyarova et al., 2007; Albacete et al., 2008; Ghanem et al., 2008; Nishiyama et al., 2011). In agreement with above observations we found that all CK HB genes up-regulated in present study (16 in blast and 11 in drought) were glucosyltransferases, which are known to be involved in conversion of active CKs to inactive conjugates (Table S1).

**Figure 4:**
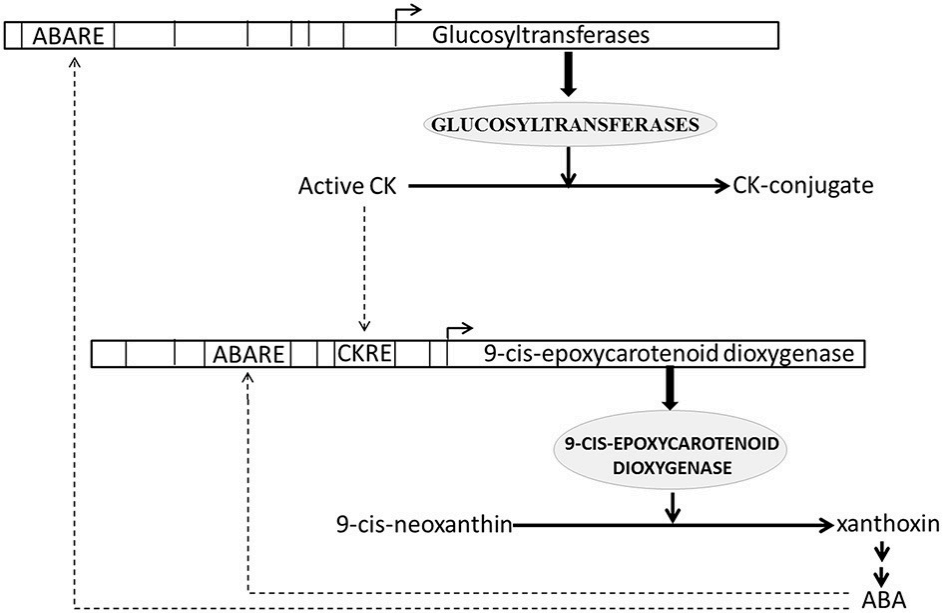
**Schematic representation of antagonistic interaction between cytokinin (CK) and abscisic acid (ABA) biosynthesis pathways**. CK response element (CKRE) and ABA response element (ABARE) are present in promoter of 9-cis-epoxycarotenoid dioxygenase, a key enzyme of ABA biosynthesis pathway. These response elements may regulate ABA biosynthesis in response to CK and ABA signals. Conversely ABARE present in promoter of Glucosyltransferases, enzymes responsible for CK-conjugation, may modulate CK-conjugation in response to ABA signaling. CK conjugation leads to CK inactivation. Solid lines depict biosynthesis of hormones and responsible enzymes. Dashed lines depict regulation of hormone biosynthesis genes through CRE elements.

We estimated and analyzed over-represented CREs in the promoters of all up-regulated CK HB loci. We found that 3 (out of 11) and 8 (out of 16) CK HB loci in drought and blast infected leaves were also associated with various ABA response CREs respectively (Table S2). As all the up-regulated CK HB loci are involved in CK conjugation, this indicates that ABA may modulate CK conjugation associated loci to reduce active CK. This is in agreement with previous experimental studies which show ABA regulates key genes in CK biosynthesis (Takei et al., 2004; Sakakibara, 2006). Incidentally we found 9-cis-epoxycarotenoid dioxygenase 1 (*Os*07*g*05940.1), a key gene of ABA biosynthesis (Xiong and Zhu, 2003) to be up-regulated in present study as well. 9-cis-epoxycarotenoid dioxygenase converts 9-cis-neoxanthin to xanthoxin in chloroplast (Schwartz et al., 1997). Xanthoxin is then transported to cytoplasm and converted to active ABA hormone through a two step reaction (Xiong and Zhu, 2003). Thus, under both blast and drought CK conjugation and ABA production loci are up-regulated simultaneously. To dissect ABA-CK interaction further we looked for over-represented CREs in promoter of 9-cis-epoxycarotenoid dioxygenase and found a CK-response element (ARR1AT) together with five ABA response elements to be enriched. These findings suggest that there may be a crosstalk between CK and ABA HB pathway regulation (Figure 4). This corresponds well with analysis of CK mutants and regulation of CK metabolic genes, which revealed its antagonism and cross-talk with ABA (Ha et al., 2012). They also observed that CK deficient plants show increased sensitivity to ABA and up-regulation of stress and/or ABA responsive genes, whereas these effects are negated with elevation in CK levels. Hence active ABA modulates CK conjugation to reduce active CK, which in turn may lead to hypersensitivity to ABA and increased stress tolerance. Conversely active CK as well as active ABA may modulate ABA production and downstream ABA responses (Figure 4).

### 4.2. DREB2A co-ordinates ethylene and jasmonic acid biosynthesis under drought and blast attack

ET and JA interactions and their response play important role in abiotic and biotic stress management (Wang et al., 2002). In case of blast we found two ET HB genes and six JA HB genes to be up-regulated; similarly in case of drought we found two ET and four JA HB genes to be up-regulated. We compared cliques associated with these JA and ET HB loci and noted that these 7 cliques (b35:SURECOREATSULTR11 DRECRTCOREAT RAV1AAT CGCGBOXAT, b71:SURECOREATSULTR11 RAV1AAT CBFHV,d2:CBFHV LTRE1HVBLT49 ROOTMOTIFTAPOX1, d9:DRECRTCOREAT CACTFTPPCA1 GATABOX, d10:GT1GMSCAM4 CBFHV LTRE1HVBLT49, d11:POLLEN1LELAT52 CBFHV LTRE1HVBLT49, d23:CELLCYCLESC POLLEN1LELAT52 CBFHV) contain DREB protein binding elements (DRECRTCOREAT and CBFHV) under both conditions. None of dehydration-responsive elements (DREs) were found in cliques associated with up-regulated ABA loci. Moreover the transcription factor, DREB2A, that binds to these DREs (DRECRTCOREAT and CBFHV) was also found to be up-regulated under both conditions.

**Figure 5:**
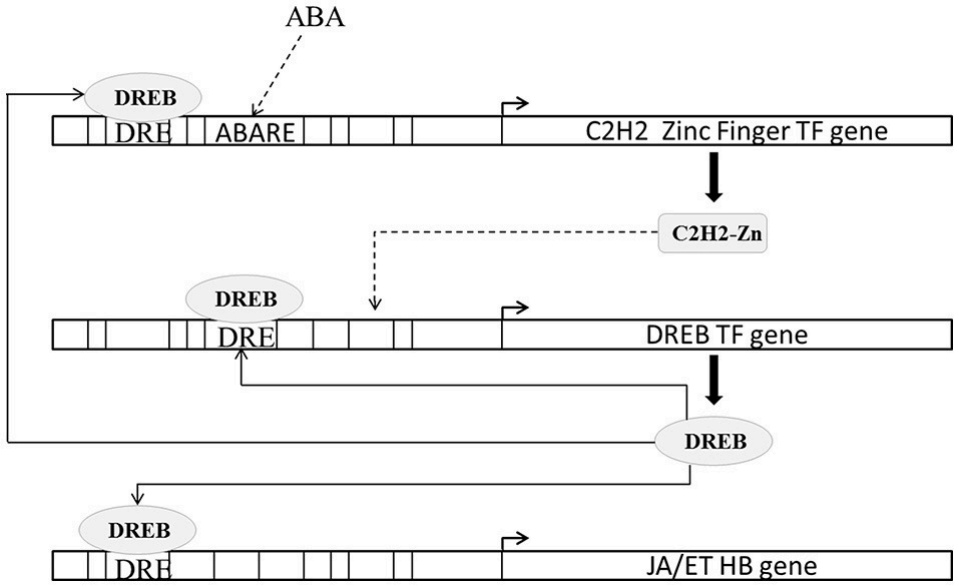
**Schematic representation of regulation of ethylene (ET) and jasmonic acid (JA) biosynthesis by DREB transcription factors**. DREB expression is regulated by C2H2 zinc finger transcription factors. C2H2 zinc finger transcription factors promoter contains abscisic acid response CRE (ABARE) and DREB binding CRE (DRE). Solid lines indicate transcriptional regulation of genes from previous literature and CRE occurrences. Dashed lines indicate transcriptional regulation of genes from CRE occurrences in present study.

DREB proteins are one of the best characterized transcription factors involved in abiotic stress response (Lata and Prasad, 2011). In rice DREB2A transcription factor (*Os*01*g*07120.2) functions are considered important under osmotic stress (Nakashima et al., 2000; Sakuma et al., 2006). DREB2A expression is induced strongly by high salt stress (Lee et al., 2010) and dehydration but weakly by exogenous application of ABA (Dubouzet et al., 2003). C2H2 zinc finger family protein (ZPF) transcription factors are known to modulate DREB expression (Figueiredo et al., 2012). ZPF179 (*Os*01*g*62190.1) and ZPF252 (*Os*12*g*39400.1) expression is known to be induced by salt and drought stress (Xu et al., 2008; Sun et al., 2010). Transgenic rice constitutively expressing ZPF179 exhibits up-regulation of DREB2A. In present study under both conditions DREB2A (3.07 fold in blast and 5.35 fold in drought) as well as ZPF179 (6.32 fold in blast and 7.72 fold in drought) and ZPF252 (2.44 fold in blast and 5.77 fold in drought) were up-regulated. However, the expressions of all three DREB2A, ZPF179, and ZPF252 were higher in case of drought than in case of blast. We found that ABA response elements were enriched in ZFP179 (ABRELATERD1, ABREOSRAB21) and ZFP252 (PROXBBNNAPA, RYREPEATBNNAPA) but not in DREB2A. DREB2A promoter contained several DREB binding CREs (DRECRTCOREA, CBFHV, DRE2COREZMRAB17) indicating that DREB2A expression may be regulated by DREB and that ABA may not modulating DREB2A directly but it may do so through ZPFs (Figure 5).

### 4.3. Cytokinin and brassinosteroids play a role in stress response and may interact directly

BR and Cytokinin are both growth promoting hormones that are known to have a role in stress management (Peleg et al., 2011; Jiang et al., 2013; Nahar et al., 2013). ABA-CK and ABA-BR are known to interact antagonistically (Tran et al., 2007; Zhang et al., 2009; Nishiyama et al., 2011). In case of blast we found six BR HB genes, sixteen CK HB genes and 9-cis-epoxycarotenoid dioxygenase (an enzyme responsible for the first committed reaction in ABA biosynthesis) to be up-regulated. In drought we found five BR HB genes, eleven CK HB genes and ABA HB gene 9-cis-epoxycarotenoid dioxygenase to be up-regulated. These results indicate that CK, BR and ABA may be important for stress response under both conditions.

BR regulates cell expansion along longitudinal axis while CK regulates cell expansion along transverse axis (Kitanaga et al., 2006). BR is known to interact with different phytohormones (Choudhary et al., 2012; Gruszka, 2013) and to promote tolerance in plants to a wide range of stresses, including heat, cold, drought and salinity, (Dhaubhadel et al., 1999, 2002; Kagale et al., 2007; Koh et al., 2007). In Arabidopsis seedlings exogenous application of BR enhanced expression of 1-aminocyclopropane-1-carboxylatesynthase (ACS), a key gene of ethylene biosynthesis pathway (Muday et al., 2012). BR also prevents 26S mediated ubiquitination and degradation of ACS protein (Hansen et al., 2009). In rice BR enhances JA level as well as promotes expression of thionin genes encoding antimicrobial peptides (Kitanaga et al., 2006). Role of CK in stress management has been recently revealed and is only partially understood. In case of rice blast disease CK levels elevated in leaf around infection site and was found to act synergistically with SA to activate PR genes (Jiang et al., 2013). However, SA levels did not alter with accumulation of CK during blast infection (Jiang et al., 2013). Rice plants over-expressing isopentyltransferase, (IPT), a key CK biosynthesis gene resulted in delayed stress response and drought tolerance. Moreover the elevated level of CKs under water stress corresponded well with decreased expression of JA biosynthesis gene OPR2 and increased expression of BR biosynthesis genes Delta14-sterol reductase (DWF5) and C-8 sterol isomerase (HYD1) (Peleg et al., 2011). ABA is known to interact antagonistically with both CK (Tran et al., 2007; Nishiyama et al., 2011) and BR (Zhang et al., 2009). The primary signaling outputs of BR are regulated by ABA signaling (Zhang et al., 2009), so whether effect of CK on BR biosynthesis is direct or mediated by ABA is not clear (Peleg et al., 2011).

**Figure 6:**
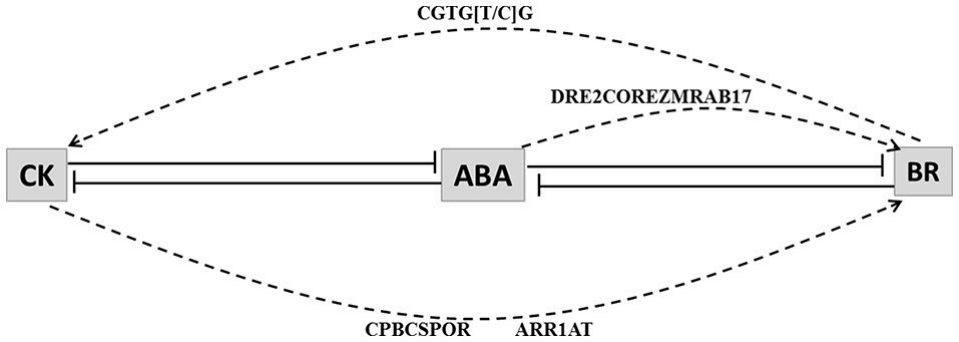
**Model depicting interaction among cytokinin (CK), abscisic acid (ABA) and brassinosteroid (BR)**. Lines with bars represent antagonistic interactions. Solid lines depict signal network interaction and dashed lines depict regulation of hormone biosynthesis genes through CRE elements. CRE elements that may be responsible for the interactions are indicated above the arrows. Abscisic acid (ABA) interacts antagonistically with both cytokinin (CK) and brassinosteroid (BR). CK response elements (CPBCSPOR and ARR1AT) and ABA response element (DRE2COREZMRAB17) are enriched in promoters of BR biosynthesis associated genes. BR response element (CGTG[T/C]G) is present in promoters of CK biosynthesis associated genes.

CK and BR HB loci promoters were inspected for known ABA (CBFHV, DRE, ABRE, DPBF, LTRE) and CK (CPBCSPOR, ARR1AT) response elements. As BR-response elements are not present in PLACE database we scanned all the CK and BR HB up-regulated loci promoters for the presence of BR response element (CGTG[T/C]G) (Reinhold et al., 2011). BR response element was found it to be present in 3 BR loci and 2 CK loci in drought condition; and in most of CK and BR loci under blast attack. In promoters of up-regulated BR HB genes *Os*05*g*29990.1 (NAD dependent epimerase) and *Os*08*g*17500.1 (cinnamoyl-CoA reductase), CK (CPBCSPOR, ARR1AT) response elements were present. These loci are associated with initial steps of BR biosynthesis. *Os*08*g*17500.1 and another loci with similar annotation *Os*02*g*08420.1 were also associated with ABA responsive element DRE2COREZMRAB17. Therefore, our results indicate that in case of blast CK and ABA both may interact directly with BR HB loci likewise BR may also interact directly with CK HB loci (Figure 6).

### 4.4. Salicylic acid and jasmonic acid are unlikely to effect each-others biosynthesis directly

SA and JA are stress hormone, SA is related to plant defense against pathogen attack whereas JA response is associated with both abiotic and biotic stress. Promoters of SA and JA HB loci were found to contain several common CREs (ASF1MOTIFCAMV, CGCGBOXAT, SURECOREATSULTR11, WRKY71OS, ACGTCBOX, CTRMCAMV35S). These common CRE were associated with 72.4 % of cliques (combination of CREs) for blast infection. These observations indicate that JA and SA HB loci may be co-regulated under fungal attack. Previous research has shown that interactions between SA and JA are complex and condition specific (Thaler et al., 2012), however antagonistic interactions seem to prevail (Pieterse et al., 2009; Tsuda et al., 2009). Cross-talk between SA and JA is proposed to play a role in fine-tuning of plant defense response (Pieterse et al., 2012). In a study expression of thirteen OPR (12-oxo-phytodienoic acid reductase—enzyme that converts linolenic acid to jasmonic acid) isogenes was monitored under different stress conditions. It was found that OPR1, OPR2, OPR10, and OPR13 in rice were induced by SA (Jang et al., 2009). SA may regulate JA biosynthesis through intermediaries i.e., NPR1 or directly.

NPR1 is known to play an important role in SA-JA antagonism, NPR1 is required for functional salicylate signaling in Arabidopsis-Pseudomonas syringae pathosystem. When NPR1 is impaired through a mutation, it resulted in enhanced expression of LOX2, an important enzyme in JA biosynthesis (Spoel et al., 2003). We also found NPR1 to be up-regulated (fold change = 2.949) under blast infection. NPR1 is known to regulate expression of genes by enhancing DNA binding of TGA transcription factors (Després et al., 2003). We did find TGA binding CRE (ASF1MOTIFCAMV) in promoters of both SA and JA HB loci but TGA expression was not up-regulated under present condition. Thus, SA through NPR1 facilitated TGA binding to ASF1MOTIFCAMV may regulate SA and JA biosynthesis. However, either this does not require up-regulation of TGA or the time point, experimental conditions and data analysis strategy used for this study were inadequate to capture expression of TGA.

JA HB genes LOX and four OPR were found to be up-regulated but none of them had any SA-responsive CRE in their promoters. Similarly out of three only one SA HB gene *Os*04*g*43800.1 contained one JA responsive CRE (T/GBOXATPIN2). *Os*04*g*43800.1 is a phenylalanine ammonia-lyase which is also associated with secondary metabolite phenylpropanoid derivative synthesis. Analysis of CRE in up-regulated JA and SA HB genes under both drought and blast revealed no CRE (except one JA responsive CRE in SA loci) that can be tied to SA or JA response directly, so it is highly unlikely that these hormones directly affect each-other's biosynthesis.

## 5. Conclusion

Our investigations into CRE-mediated regulation of differentially up-regulated hormone biosynthesis associated loci under blast infection and drought stress revealed that hormone biosynthesis pathways are subjected to hormone cross-talk. Plant response to blast and drought is associated with transcriptional modulation of biosynthesis genes of several hormones and these hormones modulate bio-availability of each other through a complex regulatory network. Abscisic acid and Brassinosteroid regulates Cytokinin by modulating its conjugation. Conversely Brassinosteroid biosynthesis is effected by both Abscisic acid and Cytokinin. Through our analysis we found that DREB transcription factors may be important in modulation of both Jasmonic acid and Ethylene biosynthesis. Moreover we found that though DREB response are known to be ABA independent but at least DREB2A response may be regulated by ABA signaling through C2H2 zinc finger protein transcription factors. Interestingly we found no indication of direct interaction of Jasmonic acid or Salicylic acid with each other's biosynthesis pathways. Thus, multiple hormones may modulate several hormone biosynthesis pathways through a complex regulatory networks, where biosynthesis of one hormone is effected by several other contributing hormones.

## Author contributions

AD, RG, and SK conceived the idea and designed the study. AD and RG collected the data. AD developed and executed computational pipelines and took part in result analysis. RG performed literature survey and result analysis. AD and RG wrote the manuscript. SK edited the manuscript and supervised the study. AD and RG contributed equally to this study. All authors read and approved the manuscript.

### Conflict of interest statement

The authors declare that the research was conducted in the absence of any commercial or financial relationships that could be construed as a potential conflict of interest.

## Supplementary material

Table S1 **List of hormone biosynthesis associated loci with fold change >2 and adjusted ***p***-value < 0.05 for ***Magnaporthe oryzae*** infection and drought stress in rice leaves**.

Table S2 **List of cytokinin biosynthesis associated loci, their known annotations, CREs found to be enriched in their promoters, ABA responsive CREs if any present among the enriched CREs and corresponding transcription factors**.

Figure S1 **Bar graph representation of different cliques and associated loci. (A)** Cliques of CREs associated with hormone biosynthesis (HB) genes up-regulated under *Magnaporthe oryzae* infection. **(B)** Cliques of CREs associated with hormone biosynthesis genes up-regulated under drought stress. Horizontal axis represents the cliques and vertical axis represents number of loci a particular clique is associated with. Loci belonging to different HB pathways are represented by different colors. Hormone abbreviations: CK, cytokinin; SA, salicylic acid; JA, jasmonic acid; ET, ethylene; ABA, abscisic acid ; GA, gibberellic; BR, brassinosteroid.
