# Supplementary figures and images for: Regulatory Cross-Talks and Cascades in Rice Hormone Biosynthesis Pathways Contribute to Stress Signaling
Source: Front Plant Sci. 2016 Aug 26;7:1303. doi: 10.3389/fpls.2016.01303 (PMC4999436; doi:10.3389/fpls.2016.01303)

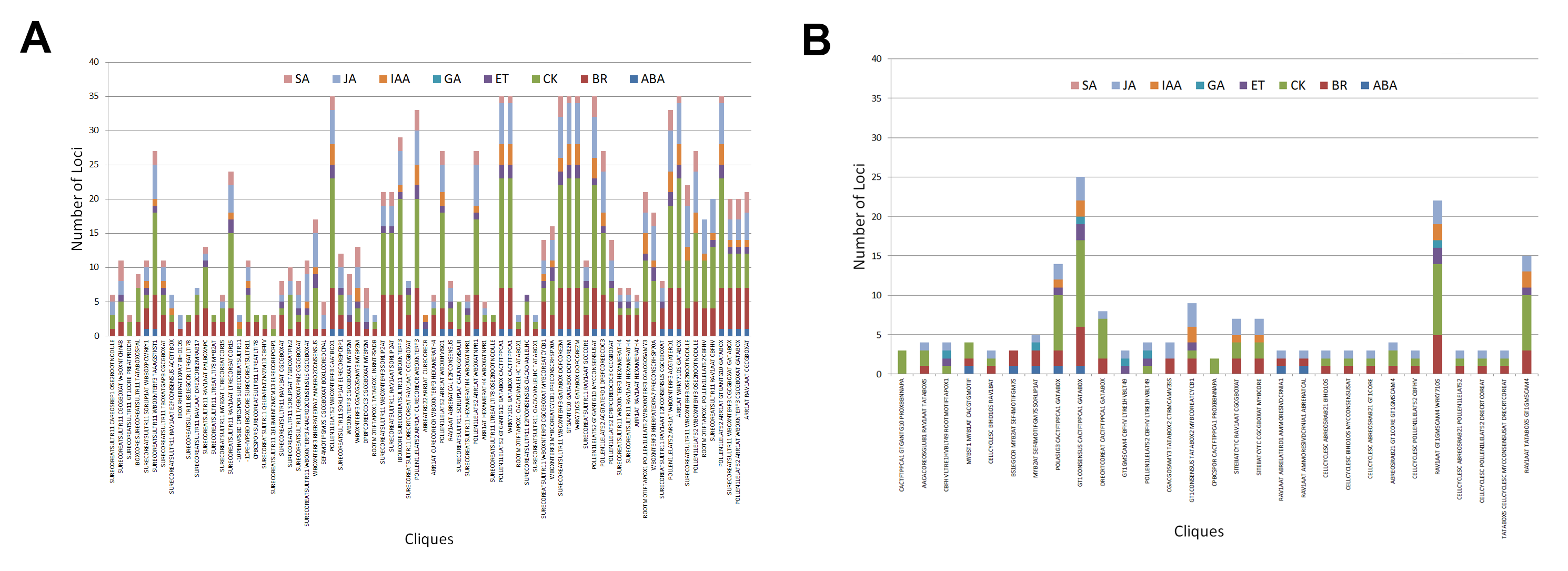

Supplement: Figure S1 — Bar graph representation of different cliques and associated loci. (A) Cliques of CREs associated with hormone biosynthesis (HB) genes up-regulated under Magnaporthe oryzae infection. (B) Cliques of CREs associated with hormone biosynthesis genes up-regulated under drought stress. Horizontal axis represents the cliques and vertical axis represents number of loci a particular clique is associated with. Loci belonging to different HB pathways are represented by different colors. Hormone abbreviations: CK, cytokinin; SA, salicylic acid; JA, jasmonic acid; ET, ethylene; ABA, abscisic acid ; GA, gibberellic; BR, brassinosteroid. [file Image1.PNG]
